# Fast and Simple LC-MS/MS Method for Rifampicin Quantification in Human Plasma

**DOI:** 10.1155/2019/4848236

**Published:** 2019-02-03

**Authors:** Žane Temova Rakuša, Robert Roškar, Anita Klančar Andrejc, Tina Trdan Lušin, Nataša Faganeli, Iztok Grabnar, Aleš Mrhar, Albin Kristl, Jurij Trontelj

**Affiliations:** ^1^University of Ljubljana, Faculty of Pharmacy, Ljubljana, Slovenia; ^2^Valdoltra Orthopaedic Hospital, Ankaran, Slovenia

## Abstract

A simple, fast, and cost-effective LC-MS/MS method for quantification of rifampicin in human plasma was developed and fully validated. The plasma samples containing rifampicin and isotopically labelled internal standard rifampicin D8, were cleaned up using a Captiva ND Lipids filtration plate. Chromatographic separation was achieved on an 1290 Infinity liquid chromatograph coupled to 6460 Triple Quadrupole operated in positive mode on a core-shell Kinetex C18 column (50 × 2.1 mm, 2.6 *μ*m) by gradient elution using 0.1% formic acid in water and acetonitrile as a mobile phase. The proposed method is the fastest method published by now, both in terms of sample preparation (approximately one minute per sample) and chromatographic analysis (total run time 2.4 min). Another key benefit is the outstanding sensitivity and wide analytical range (5-40000 *μ*g/L) with good linearity, accuracy, and precision. The method showed almost complete recovery (92%) and absence of any significant matrix effect as demonstrated by uniform responses from QC samples prepared in blood plasma from 6 volunteers (RSD <5%). The proposed method was successfully applied to rifampicin quantification in 340 patients' plasma samples, thus demonstrating its suitability for both therapeutic drug monitoring and pharmacokinetic analysis.

## 1. Introduction

Tuberculosis is an aggressive infectious disease caused by Mycobacterium tuberculosis. According to WHO, it is currently the leading cause of death from a single infectious agent, with a reported mortality of 1.6 million in 2017 [1]. Rifampicin (RIF) is highly effective bacteriostatic and bactericidal macrocyclic antibiotic, primarily used as one of the first line drugs in the treatment of tuberculosis [2]. It is also indicated in the treatment of leprosy, meningitis, osteomyelitis, and staphylococcal endocarditis [3]. Furthermore, the role of RIF is well-defined in patients with prosthetic joint infections [4]. RIF pharmacokinetics has been reported as complex with significant interindividual variability and plasma concentrations below the recommended therapeutic range (8000-24000 *μ*g/L) in majority of adult patients [5–8]. The latter is tightly associated with therapeutic failure and development of drug resistance. Furthermore, it has been reported that the pediatric population differs in RIF pharmacokinetic parameters, requiring almost twofold higher dosage in mg/kg body weight, compared to adults to reach equivalent plasma concentrations [5]. Considering its pharmacokinetic variability, therapeutic drug monitoring (TDM) is a useful and valuable tool for improvement of RIF effectiveness by individual dose adjustment and consequently prevention of therapeutic failure and drug resistance [9]. RIF TDM may be also beneficial in the sense of preventing or reducing the incidence of potential toxicity and side effects, including hepatotoxicity, which is the most common serious adverse effect, reported in 13-36% of the patients [10]. RIF determination in patients' plasma is further recommended in multidrug therapies, since it is a potent inducer of drug transporters and metabolizing enzymes. As such, RIF may interact and thus reduce the plasma concentration of other drugs, including other tuberculosis drugs, which are often used in combination with RIF [11].

For routine monitoring of RIF plasma concentration a simple, selective, robust, and cost-effective method is needed. There are several reported methods for RIF quantification in human plasma, based primarily on HPLC-UV [12–24] and LC-MS/MS [9, 10, 25–33]. However, all of them are associated with certain disadvantages to their widespread use in clinical practice. The most commonly encountered issues include lengthy (≥20 min) and/or complex extraction procedures [9, 12–19, 21–24, 28, 30, 31] and relatively long analysis run time (≥10 min) [12–19, 21–24, 27, 31], making them less suitable for routine use. HPLC-UV methods are, in general, less sensitive and require relatively large sample volumes (≥200 *μ*L) [12–19, 21, 23, 24], limiting their applicability in pediatric studies. Such methods are applicable for TDM of RIF, but not for its pharmacokinetic studies due to insufficient limits of quantification [12, 13, 15–19, 21–24]. Sufficient sensitivity and efficiency can be achieved with methods based on LC-MS/MS. However, the reported LC-MS/MS methods are mostly associated with lack of isotopically labelled internal standard [9, 28–31], persistent carry-over effect [29], and inadequately implemented matrix effect determination, mostly in terms of lack of relative matrix effect determination [9, 10, 25, 26, 28–31].

Therefore, the aim of this work was to develop and validate a simple, fast, and sensitive LC-MS/MS method for rifampicin quantification in human plasma. The proposed method is undoubtedly appropriate for routine use in both TDM and pharmacokinetic studies, which was confirmed on 340 samples from 56 different patients.

## 2. Experimental

### 2.1. Chemicals and Reagents

Rifampicin (>97.0%) was purchased from Fluka, Honeywell (Morris Plains, NJ, USA). Stable-isotope labelled rifampicin D8, used as internal standard (IS) was purchased from Alsachim (Illkirch-Graffenstaden, France). HPLC grade methanol and acetonitrile, as well as LC-MS grade acetonitrile, were obtained from Honeywell (Morris Plains, NJ, USA). Formic acid was obtained from Merck (Darmstadt, Germany). Milli-Q water was generated by an Advantage A10 water purification system (Millipore Corp., Billerica, USA).

### 2.2. Instrumentation and Chromatographic Conditions

The pretreated samples were analyzed by an Agilent 1290 Infinity liquid chromatograph coupled to 6460 Triple Quadrupole detector (Agilent Technologies, Santa Clara, USA) equipped with a Jetstream electrospray interface. The chromatographic separation was achieved on a core-shell Kinetex C18 column (50 × 2.1 mm, 2.6 *μ*m) (Phenomenex, Torrance, USA) at 50°C, by gradient elution using 0.1% formic acid in Milli-Q water (mobile phase A) and 99.8% acetonitrile, 0.2% Milli-Q water (mobile phase B) with the following gradient (min, % B, flow rate in mL/min): (0.0, 30, 0.800), (0.25, 35, 0.750), (0.5, 45, 0.650), (1.0, 55, 0.500), (1.25, 65, 0.500), (1.5-1.7, 95, 0.800), (1.9,30, 0.900), and (2.00, 30, 0.800). The injection volume was 1 *μ*L. Total run time including reequilibration was 2.4 min. The mass spectrometer was operated in positive mode using the following parameters of the ion source: gas flow (and temperature): 5 L/min (275°C), nebulizer pressure: 310 kPa, and sheath gas flow (and temperature): 11 L/min (400°C), and the capillary voltage was 4000 V. The fragmentor voltage was 80 V. Both quadrupoles were set at the widest mass resolution (2.5 amu) with the following* m/z* transitions: 823.4 → 107.1 at 61 eV and 823.4 → 163.1 at 41 eV for qualifier and quantifier ion of rifampicin, respectively. For IS, the* m/z* transition 831.5 → 105.2 at collision energy of 85 eV was used. Instrument control and data acquisition and processing were performed with Mass Hunter Workstation software B.06.00 (Agilent Technologies, Santa Clara, USA).

### 2.3. Preparation of Calibration Standards and Quality Control Samples

Individual stock solutions of IS and RIF (primary stock solution, PS) with concentration 1000 mg/L were prepared by dissolving appropriate amounts in methanol. RIF secondary, tertiary, and quaternary stock solutions were prepared with appropriate PS dilutions with a mixture of methanol and water (1:1, v/v) to obtain solutions with 500, 50, and 5 mg/L, respectively. These four stock solutions were further diluted with the same solvent to obtain eight working solutions in the range 0.1-800 mg/L. Aliquots (50 *μ*L) of the individual working solutions were diluted 20-fold with blank plasma to obtain calibration standards with the following RIF concentrations: 5, 25, 50, 100, 1000, 5000, 15000, and 40000 *μ*g/L. Quality control (QC) samples were prepared on six levels (15, 75, 750, 3000, 10000, and 30000 *μ*g/L) from freshly prepared RIF PS, following the same protocol as for calibration standards.

The extraction procedure combined protein precipitation with solid-phase extraction (SPE) and was as follows: a 100 *μ*L aliquot of plasma sample was mixed with 300 *μ*L of ice cold acetonitrile containing IS (2.5 mg/L). After precipitation, the sample mixture was filtered through the Captiva ND Lipids filtration plate (Agilent Technologies, Santa Clara, USA) using vacuum manifold. The collected filtrates were transferred to the autosampler and subjected to LC-MS/MS analysis. Unless otherwise noted, RIF concentration was determined based on RIF/IS ratio.

### 2.4. Sample Collection and Preparation

A significant number of blood samples (340) were obtained from 56 patients, receiving 450 mg RIF every 12 hours. The study was approved by the Republic of Slovenia National Medical Ethics Committee (approval no. 48/06/11). Samples were collected at different predetermined time points schedule according to the site of the infection and were properly treated to obtain plasma samples. Blank plasma samples were obtained from six healthy volunteers. Plasma samples were stored at -80°C and thawed prior to analysis. The sample preparation procedure was exactly the same as for the calibrator and QC samples. All patient samples were processed in at least two parallels.

### 2.5. Method Validation

The proposed method was validated according to the EMA guideline on bioanalytical method validation [34]. Recovery, relative, and absolute matrix effect were evaluated in accordance with Matuszewski et al. [35, 36].

Method selectivity was assessed by the analysis of plasma samples from six different lots, which were evaluated for interference with RIF or IS. Furthermore, RIF identity was confirmed when the deviation of qualifier to quantifier ion ratio was less than 20%.

The lower limit of quantification (LLOQ) was determined as the lowest calibration standard, which reached acceptable accuracy (100 ± 20%) and precision (RSD below 20%). Additionally, the RIF signal in LLOQ was checked to be at least 5-fold greater compared to the response from blank sample.

Method linearity was evaluated on eight nonzero calibration standards in the concentration range from 5 to 40000 *μ*g/L for three consecutive days of the validation. Linearity was determined based on the least-square linear regression. The acceptance criterion was a correlation coefficient of more than 0.99.

Carry-over was assessed by injecting blank sample after the highest calibration standard (40000 *μ*g/L) and high concentration samples. The acceptance criterion was carry-over of less than 20% of the LLOQ and 5% for the IS.

Accuracy and precision of the method were determined on QC samples at six concentration levels covering the calibration range and were prepared fresh on each of the three consecutive days of the validation. Within-run accuracy and precision were evaluated on QC samples prepared in five replicates and analyzed in a single run. Between-run accuracy and precision were evaluated on QC samples (n=6) prepared and analyzed on each of the three consecutive days of the validation. Accuracy was acceptable when the mean concentration was within ± 15% of the nominal value for all QC samples, except at the LLOQ, where ± 20% deviation was accepted. Acceptance criterion for precision, expressed as the coefficient of variation (CV), was ± 15% for the QC samples, except at the LLOQ (± 20%).

Recovery, absolute, and relative matrix effect were evaluated on four QC levels (15, 750, 10000, and 30000 *μ*g/L) each prepared in triplicate. Relative matrix effect (RME) was determined on QC samples prepared from blank plasma from six individual donors and was expressed as CV (%) of RIF responses at each concentration level. CV (%) value of standard line slopes obtained from six different plasma lots was also calculated. Absolute matrix effect (AME) was determined at each QC concentration level as the ratio between blank plasma spiked after extraction with RIF and IS (B) and standard RIF and IS solution in neat solvent (A): AME = B/A × 100%. Recovery (RE) was determined as the ratio of RIF responses of extracted QC samples and blank plasma spiked after extraction with the same nominal RIF and IS concentration as in the QC samples (B): RE = QC/B × 100%.

RIF stability was evaluated on four QC levels (15, 750, 10000, and 30000 *μ*g/L), each prepared in triplicate. Stability studies included freeze and thaw stability (after three cycles), short-term stability (after 4h at room temperature) of RIF in human plasma, and autosampler stability of RIF in extracted samples (reanalyzed after 24 hours at 4°C). Deviations in mean RIF concentration at each QC level of less than 15% were considered acceptable.

## 3. Results and Discussion

### 3.1. Method Optimization

Mass spectrometry ion source conditions were optimized to achieve the highest possible responses for both RIF and its isotopically labelled IS (RIF-D8). The optimal MRM transitions were obtained by automated method development software (MassHunter Optimizer, Agilent Technologies). The most abundant fragment ion was chosen as quantifier, and the second most abundant as qualifier; the ratio of qualifier to quantifier ions was used to confirm the RIF identity. For IS, only one mass transition was used in order to increase the sensitivity of the method towards RIF.

The chromatographic method utilizing a core-shell C18 column was optimized to provide fast and robust RIF separation from background matrix with low system back-pressure, making it suitable not only for UHPLC but for ordinary HPLC systems as well.

Sample preparation procedure for RIF quantitation was based on a method described by Srivastava et al. [31], which was further optimized in terms of substantial time reduction, which is especially important when large sample batches need to be analyzed. Based on literature findings [37], as well as previous practical experience with plasma protein precipitation, acetonitrile was chosen as the optimal organic solvent. Typical plasma samples preparation procedures include vigorous vortex mixing to allow protein precipitation, followed by centrifugation to remove the precipitates. These conventional and most time consuming steps in bioanalysis were upgraded to in-well protein precipitation and cleanup by Captiva ND Lipids filtration plate. With the introduction of this fast and simplified workflow, sample preparation time was reduced from approximately 30 min to 1 minute per sample, with high recovery and lipid matrix removal. Such sample preparation is cost-effective and can be fully automated for high-throughput sample analysis.

### 3.2. Method Validation

**Figure 1:**
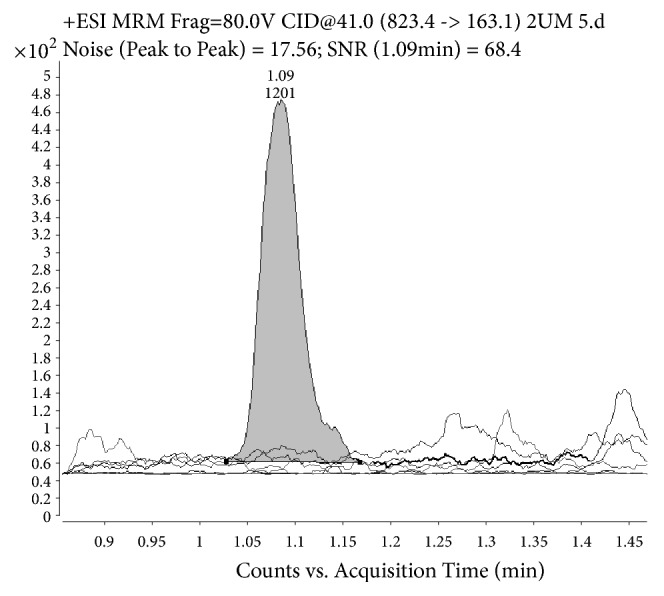
Representative LC-MS/MS chromatograms of six blank plasma samples and LLOQ sample. Retention time of RIF is 1.1 min.

Method selectivity was confirmed as no interference from endogenous compounds was observed at the retention time of RIF or IS in the individual blank plasma from six different sources. Representative chromatograms of six blank plasma samples and LLOQ sample are presented in Figure 1. Rifampicin qualifier to quantifier ion intensity ratio (average 0.69) was within the acceptance criterion (± 20%) in all tested samples.

Linearity of the method was confirmed over a very wide concentration range: 5-40000 *μ*g/L, which completely covers the therapeutic range of rifampicin (8000-24000 *μ*g/L). The obtained calibration curve from the first validation day, plotted as response ratio of RIF to IS on y-axis versus RIF concentration on the x-axis of using weighting of 1/c, was y=0.003220x-0.004509. The corresponding determination coefficient (r^2^) was 0.9993. The method's LLOQ was the lowest calibration standard (5 *μ*g/L) based on precision, accuracy, and LLOQ/blank sample signal ratio. The method was therefore found sufficiently sensitive for RIF determination in bioequivalence and pharmacokinetic studies. The wide concentration range enables RIF quantification in various samples without the need of any additional sample processing (e.g., dilution).

Carry-over effect has previously been reported as a troublesome part of RIF LC-MS/MS analysis [29, 38]. However, the analysis of blank solutions immediately after the highest calibration standard and high concentration samples revealed no quantifiable carry-over.

The obtained results for within-run and between-run accuracy and precision are within the acceptance criterion, as presented in Table 1.

The evaluation of recovery and matrix effect is a very important part of bioanalytical LC-MS/MS method validation. It is, however, somewhat neglected or undervalued aspect, due to incomplete experimental protocol by the FDA and EMA guidelines in terms of relative matrix effect evaluation [34, 39]. The latter is a crucial parameter in bioanalytical assays validation. The greatest drawback of previously reported methods is RIF quantification in human plasma from different patients, without appropriate demonstration that the relative MS/MS response is not affected by the matrix. Elimination of relative matrix effect uncertainty is, thus, essential in order to obtain reliable and useful results for further pharmacokinetic analysis. Therefore, relative matrix effect was quantitatively assessed as proposed by Matuszewski et al. [35]. The obtained results for relative matrix effect (CV <5%) in combination with the low CV of standard line slopes obtained from six different plasma lots (Table 2) are an excellent demonstration of the absence of relative matrix effect. The obtained average absolute matrix effect ± SE for RIF/IS ratio was 101.89 ± 1.95%. Based on the obtained results for both relative and absolute matrix effect, it can be concluded that the proposed method is free from any major ion suppression or enhancement. The mean recoveries (RE ± SE) for both RIF (92.48 ± 3.97%) and IS (88.39 ± 3.07%) were consistent and reproducible, thus confirming the suitability of the extraction procedure.

The results for freeze and thaw stability, short-term stability, and autosampler stability, expressed as % of change from the initial sample, are summarized in Table 3. The stability of RIF in all QC samples was acceptable (± 15% change) under all tested conditions. Long-term stability was not assessed since RIF has previously been shown stable in plasma stored at -20°C for at least 1 month [9] and for 4 months after storage at -80°C [2]. Patients' plasma samples were stored at -80°C and were analyzed as soon as possible.

### 3.3. Application and Prospect of the Method

**Figure 2:**
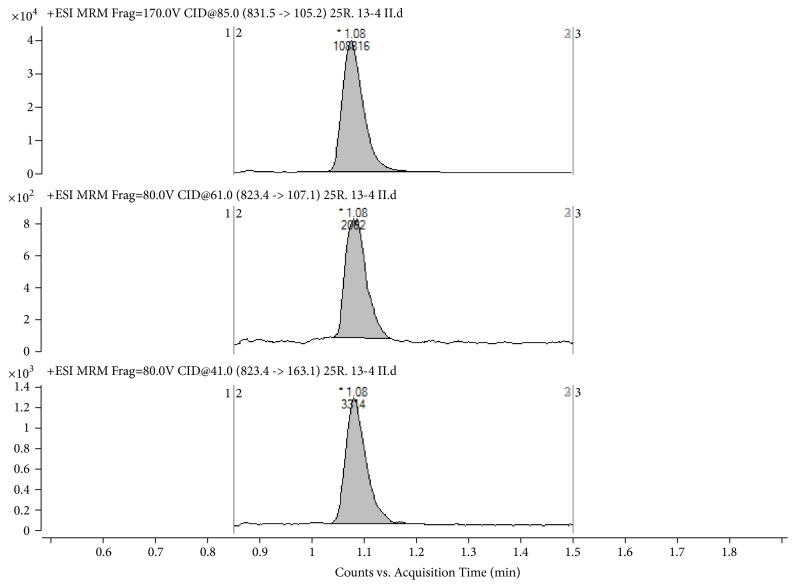
LC-MS/MS chromatogram of a real plasma sample from a patient, receiving 450 mg RIF every 12 hours. The determined RIF concentration is 28.3 *μ*g/L. Top trace represents IS; middle trace represents RIF qualifier ion, and bottom trace represents RIF quantifier ion.

The applicability of the proposed method was confirmed on 340 plasma samples from 56 patients with orthopedic endoprosthesis infections, receiving 450 mg RIF every 12 hours. The determined RIF concentrations ranged between 5.92 and 21447 *μ*g/L. Calculated mean RIF concentration (with standard deviation) in the assayed samples with RIF concentrations above LLOQ was found 2433 ± 3324 *μ*g/L. An example of a real plasma sample chromatogram, with determined RIF concentration 28.3 *μ*g/L, is shown in Figure 2. The analysis of such a number of plasma samples was facilitated by the fast and simple sample preparation, which took approximately one minute per sample. Sample preparation time can be further reduced by the use of a fully automated approach. This, in combination with the fast LC-MS/MS method with total run time of 2.4 min, is the most important advantage compared to other reported methods for RIF quantification. Additional advantages include low LLOQ and wide analytical range, making it suitable for routine analysis of plasma samples with diverse RIF concentrations. Due to the small plasma volume (100 *μ*L), the proposed method is also suited for pediatric studies, where sample volumes are commonly quite limited. This assay is therefore undoubtedly applicable for pharmacokinetic, bioequivalence, or bioavailability studies of RIF, as well as for TDM.

## 4. Conclusions

The developed LC-MS/MS method was fully validated in terms of selectivity, linearity, accuracy, precision, dilution integrity, carry-over, recovery, matrix effect, and stability. Its suitability for routine analyses was confirmed by rifampicin quantification in 340 patients' plasma samples. The fast, simple, and efficient sample preparation, along with the short LC-MS/MS run time, enables the analysis of a large number of plasma samples in a short time, thus providing a fast, reliable, and cost-effective analytic tool for RIF therapeutic monitoring and pharmacokinetic studies.

## Figures and Tables

**Table 1:** Accuracy and precision data for RIF.

QC (*μ*g/L)	Accuracy (%)		Precision (CV%)	
	Within-run	Between-run	Within-run	Between-run
15	105.04	100.47	1.95	6.70
75	99.23	97.93	1.25	3.95
750	87.21	88.67	0.63	7.92
3000	98.43	98.09	1.27	2.35
10000	98.23	101.80	3.52	6.43
30000	103.87	106.45	2.97	5.32

**Table 2:** Relative matrix effect data for RIF.

	Relative matrix effect (CV%)	
QC (*μ*g/L)	IS correction	Without IS correction
15	4.75	12.00
750	2.97	13.34
10000	3.26	11.03
30000	3.65	2.66

slope	2.71	2.94

**Table 3:** Stability data for RIF.

	Stability (%)		
QC (*μ*g/L)	Freeze and thaw	Short-term	Autosampler
15	3.03	5.06	-9.88
750	-10.23	-1.89	N.D.
10000	3.00	-3.80	N.D.
30000	1.61	2.24	-2.18

N.D. = not determined

## Data Availability

The data used to support the findings of this study are included within the article.
